# Phenology and Floral Biology of *Diospyros sericea* A. DC. (Ebenaceae): Inconstant Males May Be behind an Enigma of Dioecy

**DOI:** 10.3390/plants11192535

**Published:** 2022-09-27

**Authors:** Bárbara Ramaldes, Renata Santos, André Rodrigo Rech, Michellia Soares

**Affiliations:** 1 Botany Laboratory, Alegre Campus, Federal University of Espírito Santos, Alegre 29500-000, ES, Brazil; 2 Laboratory of Forest Seeds, Department of Silviculture and Forest Genetics, Federal University of Lavras, Lavras 37200-900, MG, Brazil; 3 Centre of Advanced Studies on Ecological Systems and Interactions, JK Campus, Federal University of Jequitinhonha and Mucuri Valley, Diamantina 39100-000, MG, Brazil; 4 Laboratory of Plant Ecology and Systematics, Federal Institute of Northern Minas Gerais, Salinas Campus, Salinas 39560-000, MG, Brazil

**Keywords:** genus *Diospyros*, sexual dimorphism, reproductive system, evolution

## Abstract

*Diospyros sericea* is a tree/shrub species considered dioecious and broadly distributed in Brazil. Despite its importance for niche composition in a range of ecosystems, there is little knowledge about this species, and so far no study has analyzed its sexual system. We aimed to investigate dioecy expression in *D. sericea* through sexual dimorphisms in its phenology and floral biology. We analyzed the phenological events over a year and studied floral biology traits (morphology, flower development, floral resource, floral attractants supply, viability of pollen, and stigma receptivity) in both male and female plants. *D. sericea* presents typical features of dioecious plants like well-established primary and secondary dimorphisms that contribute to its reproductive success. However, we also identified fruit development in what should be structurally male individuals. We suggest that the evolutionary pathway leading to the observed phenomenon may be the existence of subdioecious populations with “inconstant males”. Although our data prevented us from making further assumptions about the origin of this trait, the study contributes to future analyses towards unraveling the enigma of dioecy not only in *D. sericea* but in other *Diospyros* species.

## 1. Introduction

Angiosperms are currently the most diverse plant group on earth. Their origin and the causes of their great expansion were considered by Darwin as an abominable mystery [1]. As sessile organisms, these plants depend on pollinating agents for pollen transfer [2], establishing a coevolutionary and exclusive relationship with animals [3,4]. Floral visitors directly affect the pollen flow [5]; therefore, investing in attraction resources may increase the number of fertilized ovules [6] and favor outcrossing.

Outcrossing is essential to genetic diversity [7], while selfing may pose harmful effects to subsequent generations [8,9]. Therefore, mechanisms that reduce self-pollination and allow outcrossing may have directly influenced floral evolution [2], contributing to the diversity of floral morphologies currently observed among angiosperms. 

Functional hermaphroditism is the most common sexual system among angiosperms [10,11]. In cosexual plants, it may appear in hermaphroditic plants bearing only perfect flowers (about 90% of angiosperm species) and monoecious plants in which a single individual bears both male and female unisexual flowers [2,12,13,14], among others. Dioecy, with unisexual male and female flowers in different individuals (reviewed by [7,12,13,15,16]), appears at a lower frequency of around 6 to 7% of the species [16,17].

Many hypotheses have been proposed to explain the establishment of dioecy in angiosperms. It has been demonstrated across multiple groups that dioecy has had multiple origins across evolutionary time [2,13,18]. One possibility is the evolution of dioecy from self-compatible non-dioecious ancestors [14,19], avoiding inbreeding and optimizing resource allocation. This would have involved at least two types of mutation: one that caused male infertility, producing individuals with structurally female flowers, and one (or more) mutations that suppressed female fertility, producing individuals with structurally male flowers [20,21,22,23] that occurred successively, not simultaneously. This would have involved the existence of populations with intermediate sexual types composed of some individuals bearing perfect flowers and other individuals bearing flowers with one of the organs (stamen/carpel) sterile based on the mutation, characterizing androdioecy and gynodioecy [20]. 

Current research has attempted to trace the evolutionary pathways to the existence of sex chromosomes in plants and, for this effect, sex determination has been widely studied [14,24,25]. Genetic mutations causing the sterility of reproductive functions are expected to have antagonistic and pleiotropic effects [14,26]. For example, the female sexual organs were not expressed, and/or male functions were enhanced in hermaphroditic ancestors by re-allocating reproductive resources from female to male functions, thus resulting in subdioecious populations [14]. Although normally considered dioecious, with only occasional monoecious individuals, male plants are regarded as “inconstant” due to their ability of occasional seed production [14,21]. Conversely, during this hypothetical evolutionary pathway between monoecy and dioecy, intermediate paradioecious populations could have appeared, in which a given plant, mostly bearing unisexual flowers of one sex, could bear flowers from the opposite sex [12].

The differences between female and male individuals in dioecious species are related to sexual dimorphism [10], which can occur in morphology, physiology, and life history [10,27]. Primary sexual dimorphism refers to sexual differences between the androecium and the gynoecium, whereas secondary sexual dimorphism produces morphological, physiological, and phenological differences between the sexes [12,28]. 

Dioecy is generally related to some ecological traits [15,29,30,31]. These include the occurrence at high elevations [16], generalized pollination [32], fleshy fruit production [15,30,31], and animal seed dispersal [30,33]. Molecular phylogenetic analyses have shown an association of these ecological traits with the diversification of dioecious lineages [34,35].

Dioecious species have been found to compose up to 12% of the endemic flora of the Brazilian Cerrado [30]. These species occur in open areas, are brevi-deciduous, and show irregular and barely evident reproductive phenology. The angiosperm family Ebenaceae is among the exclusively dioecious families that occur in the Cerrado. Hence, Ebenaceae is considered a pantropical family with a center of diversity in South America [36]. It is mainly composed of woody plants with a tree, shrub, or sub-shrub habit [36,37,38]. Encompassing more than 500 species, *Diospyros* L. is the most diverse genus within Ebenaceae [36,39,40]. Sixty-two species of this genus occur in Brazil and twenty-nine are endemic to the country [41].

Species of *Diospyros* have been used as models in molecular research aiming to elucidate dioecy evolution and expression. Although sex-determining genes among *Diospyros* species have been identified [7,14], studies on its reproductive biology are scarce [40]. This scarcity can be confirmed with *D. sericea* A. DC. This species is broadly distributed in South America, including Venezuela, Colombia, and Brazil [42]. However, *D. sericea* reproductive biology is completely unknown, and, although considered a dioecious species, no study has yet attempted to compare individuals of *D. sericea* of different sexes. 

Thus, here we aim to analyze dioecy expression through sexual dimorphisms in *D. sericea* by describing its phenological events and floral biology. We hope to produce data that contribute to the conservation of *D. sericea* and the interdependent ecological balance associated with it. Our work may provide a basis for future analyses on this species towards unraveling the enigma of the origin of dioecy and its establishment in the genus and angiosperms in general. 

## 2. Results

### 2.1. Phenological Analyses

#### 2.1.1. Vegetative Phenology

We observed leafing and defoliation in all individuals (13 female and 17 male) of *D. sericea* during the monitoring months. These phenological events could be considered highly synchronous but occurred at a low intensity.

**Figure 1:**
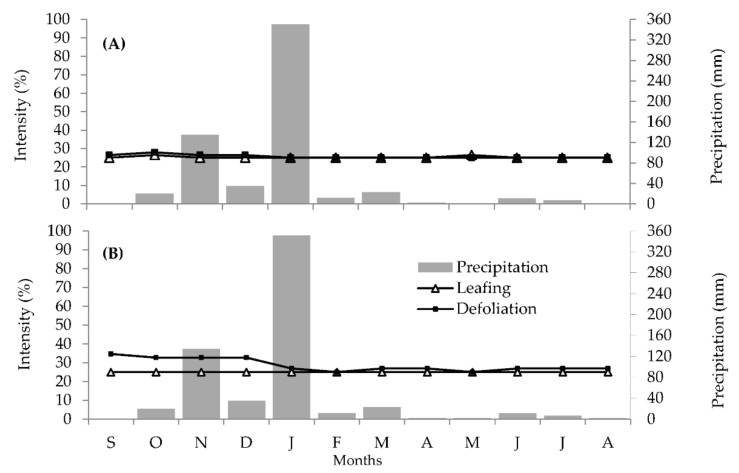
Fournier intensity of the vegetative phenology of Diospyros sericea and climatic variables in the area surrounding the Serra Nova e Talhado State Park (PESNT), district of Rio Pardo de Minas, Minas Gerais state, Brazil. Data recorded between September 2015(S) and August 2016(A). (**A**) Male individuals. (**B**) Female individuals.

Regardless of climatic variation, leafing remained constant in both male and female individuals throughout the monitored period, with ~25% intensity. Defoliation happened similarly: between 25 and 35% intensity and with little variation during the observed year (Figure 1A,B). One exception to this trend was the significant increase in defoliation that exceeded leaf production among female individuals between September and December (Figure 1B).

The Spearman correlation analysis between the vegetative phenophases and the climatic variables revealed a significant correlation between defoliation and climatic variables (temperature and humidity). Defoliation was positively correlated with temperature, peaking in the period with the highest temperature averages, and strongly negatively correlated with humidity, peaking in the periods with the lowest relative air humidity (Table 1).

Our results support the classification of *D. sericea* as an evergreen species, characterized by continuous growth (ECG), prolonged leaf production, and an absence of evident deciduousness.

#### 2.1.2. Reproductive Phenology

**Figure 2:**
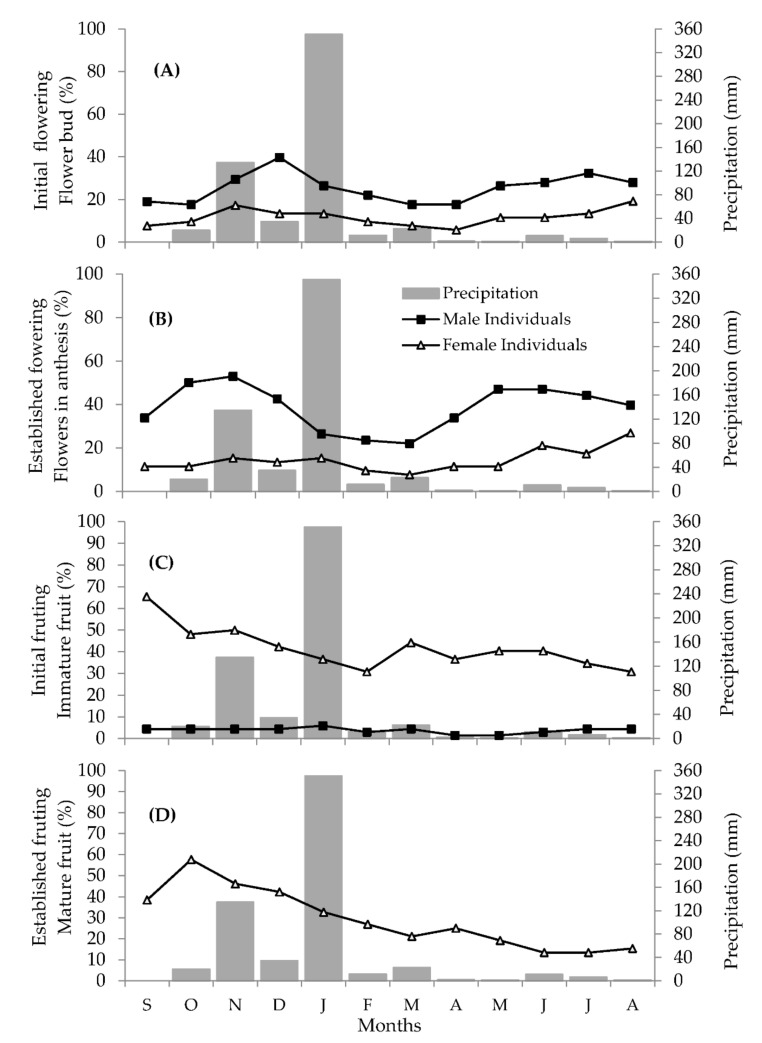
Fournier intensity of the reproductive phenology of male and female plants of *D. sericea* and accumulated precipitation (mm). Data recorded between September 2015 and August 2016. (**A**) Initial flowering intensity (flower bud); (**B**) established flowering intensity (flowers in anthesis); (**C**) initial fruiting intensity (immature fruits); (**D**) established fruiting intensity (mature fruits).

Flowering was observed from initial flowering (floral bud) to establishment (anthesis flowers) in *D. sericea* during the whole monitoring year (Figure 2A,B). However, the synchronicity of these events behaved differently among individuals of the same sex. Flowering was highly synchronous among male individuals. Among female individuals, flowering was only highly synchronous during the peak months (November, January, July to August) and remained hardly synchronous during the rest of the year.

Flowering intensity also differed between male and female populations. Flowering in male populations was twice as intense as in female populations. This trait may be connected with the number of flowers per flowering branch since male plants presented more flowers than female ones.

We observed two flowering peaks at different times. The first peak, with the highest flowering intensity, happened during the rainy season: it was first recorded among the male individuals between October and December and, later, among female individuals from November to January. The second peak, observed during the dry season between May and August, followed a similar pattern whereby flowering intensity was initially higher among the male population and was later followed by the female population between June and August. We highlight that the production of flower buds and flowers varied gradually over time among the male population, with some periods of stability. Conversely, among the female population, flowering intensity peaked rapidly and abruptly.

As with the other phenophases, fruiting was also observed throughout the monitoring period and was, for the most part, highly synchronous. However, only a single fruiting peak was observed in the transition between the dry and the rainy seasons (Figure 2C,D). The highest intensity of initial fruiting (immature fruits) was recorded in September and of mature fruits in October. We highlight that some male individuals of *D. sericea* also produced fruits (Figure 2C), although these were smaller in size, produced in lower numbers, and failed to reach maturity.

The Spearman correlation analysis revealed a general weak correlation between flowering and the climatic variables analyzed. The established fruiting (mature fruit) phenophase was the only one to show a significant correlation with climate. Mature fruit production was positively correlated with temperature and negatively with humidity (Table 1), suggesting higher fruit production under higher temperatures and lower relative air humidity.

### 2.2. Floral Biology

#### 2.2.1. Morphological Traits and Sexual Expression

**Figure 3:**
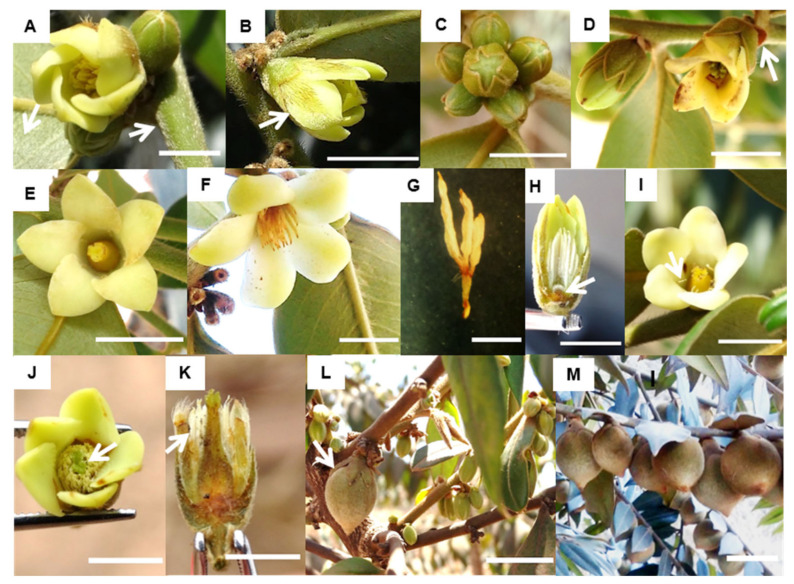
Floral biology traits of *D. sericea* located in the surroundings of the PESNT. (**A**) Sericeous aspect of the vegetative structures and (**B**) flowers, indicated by the arrow, bar = 1 cm; (**C**) multiple buds per axil in a male individual, bar = 1 cm; (**D**) solitary bud per axil in a female individual, and bracts indicated by the arrow, bar = 1 cm; (**E**) female flower with a bottle-shaped pistil, bar = 1 cm; (**F**) male flower, bar = 1 cm; (**G**) polyadelphous stamens fused at the base with free anthers, bar = 0.2 cm; (**H**) sericeous pistillode in a structurally male flower, bar = 0.5 cm; (**I**) staminodes in a structurally female flower indicated by the arrow, bar = 0.5 cm; (**J**) structurally male flower with a well-developed pistillode, with stigma indicated by the arrow, bar = 0.5 cm; (**K**) structurally female flower with well-developed staminodes indicated by the arrow, bar = 0.5 cm; (**L**) structurally male individual with a developed fruit at the branch base indicated by the arrow, bar = 3 cm; (**M**) structurally female individual with a fruit-bearing branch, bar = 3 cm.

As suggested by its name, *D. sericea* has yellow and golden pilosity that provides it a sericeous aspect. On its vegetative structures, trichomes are found along the branches and abaxial leaf surfaces (Figure 3A). On its flowers, trichomes are found on the entirely pilous sepals and the central region of the outer surface of the petals (Figure 3B).

Its sessile and downward-oriented flowers are inserted in leaf axils along the branches, covered by the abaxial surface of leaves (Figure 3A,B). Structurally male individuals have more than one flower bud per axil, forming a cymose inflorescence (Figure 3C). Female individuals showsolitary flowers (Figure 3D). 

One or two small green bracts are also observed in the floral axils. These bracts are formed along with the flower buds but quickly dehydrate (Figure 3D) and may persist after flower abscission.

Morphological similarities were observed between the male and female flowers, including color, form, symmetry, and number of perianth parts (see the comparative description of floral size below). Both the pistillate and staminate flowers are dichlamydeous, heterochlamydeous, actinomorphic, and pentamerous (Figure 3E,F), and occasionally tetramerous and hexamerous.

Both male and female flowers are inconspicuous, with a gamosepalous calyx with greenish sepals and an opaque cream-colored corolla with cyclically arranged free petals. Due to their morphological similarities, male and female flowers are only distinguishable by their reproductive whorls.

The structurally male flowers are polystaminate with a heterodynamous androecium (Figure 3F,H). The stamens are epipetalous and organized in polyadelphous bundles, each composed of three stamens attached by the filament base, with anthers pivoting freely (Figure 3G). Male flowers also have a sericeous vestigial pistillode (Figure 3H).

Structurally female flowers have a syncarpous gynoecium composed of three carpels with terminal styles that form a bottle-shaped pistil, which does not surpass the corolla height (Figure 3I). The ovary is superior and trilocular, with two ovules in each cavity. Female flowers generally have vestigial staminodes (Figure 3I). 

The pistillodes and staminodes are occasionally similar in size to those of functional pistils and stamens (Figure 3J,K). Some predominantly male individuals, with occasional hermaphroditic flowers, produced fruits. In these cases, a single individual developed several fruits, usually at the base of the branches (Figure 3L). However, these fruits failed to reach maturity and were considerably smaller than those originating from ovary development in structurally female flowers (Figure 3L,M).

#### 2.2.2. Morphometrics

Both pistillate and staminate flowers had no significant differences in the calyx and corolla lengths between the sexes, but significant differences were found in the diameter of the perianth, pistil length and diameter, and stamen length (Table 2).

The number and length of the staminodes present in the female flowers differed significantly from that of the functional stamens present in the male flowers. Among the male flowers, this was also observed in the length and diameter of the pistillodes, which differed significantly from the functional gynoecium of the female flowers. These data revealed significant structural differences in the reproductive whorls and vestigial reproductive organs of female and male flowers in D. sericea (Table 2).

The sexual dimorphism observed in the reproductive whorls reflects the significant differences found in calyx and corolla diameters between pistillate and staminate flowers. The ovary in female flowers leads to broader sepal and petal diameters. However, this was not observed among the structurally male flowers, where the pistillodes were approximately three times smaller than the pistils of structurally female flowers (Table 2). 

The number of flower buds per axil is higher in structurally male individuals (1–7 buds per axil), on average twice as many as in female individuals.

#### 2.2.3. Floral Development

**Figure 4:**
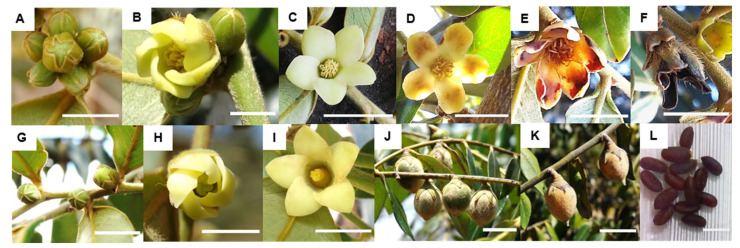
Floral development of *D. sericea* observed in the surroundings of the PESNT. Structurally male individuals: (**A**) flower buds; (**B**) flower in anthesis; (**C**) blooming flower; (**D**) flower at the onset of senescence with brownish anthers and petal lobes; (**E**) oxidation of the floral features (corolla and stamens); (**F**) completely oxidated flower. Structurally female individuals: (**G**) buds; (**H**) flower in anthesis; (**I**) blooming flower; (**J**) immature fruits; (**K**) mature fruits; (**L**) seeds. (**A**–**G**,**I**) scale bar = 1 cm; (**H**) bar = 0.5 cm; (**J**–**L**) bar = 3 cm.

Floral development begins with the formation of buds, which are initially completely covered by the sepals (Figure 4A,G). The petals display spiral flowering (Figure 4B,H). The anthesis period may last from two to three days until complete flower opening (Figure 4C,I).

Structurally male flowers remain completely open during the fourth and fifth day of floral development, when senescence begins. This process can be observed through the oxidation of the floral whorls, beginning in the anthers and petal lobes (Figure 4D), which become progressively brown (Figure 4E). From the sixth to the seventh day, the flowers become completely oxidated and dry (Figure 4F) and may suffer abscission.

In structurally female flowers, fruit development begins early, during anthesis, before the flowers are completely open. Initially, *D. sericea* berries are densely sericeous with a greenish color (Figure 4J). During maturation, the fruits become orange and some of their pilosity is lost (Figure 4K). The seeds are elongated and brown (Figure 4L). The flowers that fail to develop fruits before the completion of anthesis undergo an oxidative process that culminates in flower senescence.

#### 2.2.4. Pollen Integrity and Stigma Receptivity

**Figure 5:**
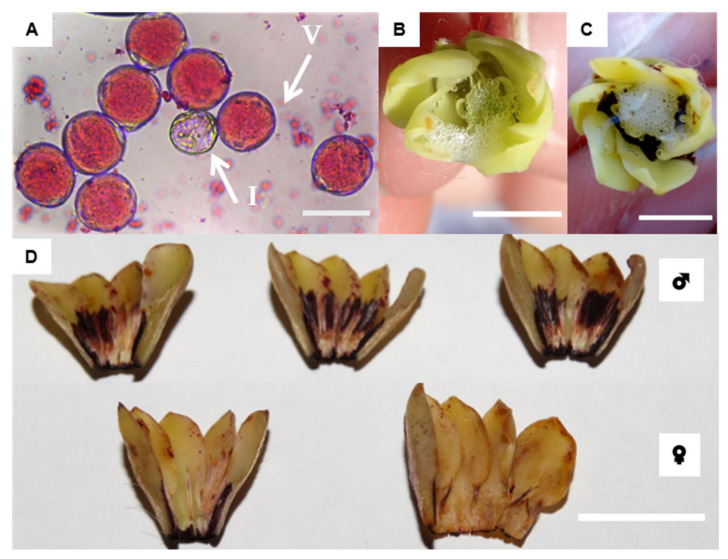
Results of the pollen integrity and stigma receptivity tests and the presence of osmophores in flowers of *D. sericea* collected in the surroundings of the PESNT. (**A**) Acetocarmine-stained pollen, V = viable, and non-stained pollen, I = inviable, bar = 50 μm; (**B**) sign of stigma receptivity from a reaction to hydrogen peroxide in a pre-anthetic flower and (**C**) in a blooming flower, bar = 0.5 cm; (**D**) neutral red-positive coloration of scent glands in the petals and stamens or staminodes: above, male flowers (♂), below, female flowers (♀), bar = 1 cm.

In individuals with structurally male flowers, the flower buds, flowers in pre-anthesis, and completely open flowers had an expressive number of intact pollens. This trait was identified by the acetocarmine reaction. Pollen is spherical, whitish, and arranged in monads, with a powdery appearance (Figure 5A).

Pollen integrity exceeded 90% in the three stages of floral development (Table 3; Figure 5A). In structurally male flowers, the percentage of pollen integrity was maintained throughout floral development. According to the analysis of variance (ANOVA), there were no significant differences in the pollen integrity between flower buds, flowers in pre-anthesis, and completely open flowers from different and random individuals (F = 0.7627; *p* = 0.5198, Table 3). On the other hand, in flowers and flower buds of female individuals, no pollen grains were detected in the staminodes. 

The stigma receptivity test conducted in flower buds, flowers in pre-anthesis, and open flowers showed positive results (Figure 5B,C). The open flowers displayed a stronger reaction than the other stages, with greater formation of bubbles than in the other stages. These data could either indicate a greater receptivity during this stage or a reaction to necrosis enzymes. We also tested receptivity in the pistillodes of structurally male flowers, but the results were always negative.

#### 2.2.5. Floral Attractants

The scent exhaled by *D. sericea* flowers is sweet and, by the human sense of smell, it is considerably more intense in male flowers than in female flowers. Scent glands (osmophores) were identified at the corolla margins and in the anther region of the stamens. The structurally male flowers showed a larger red-brownish-colored region than the female flowers (Figure 5D).

## 3. Discussion

*D. sericea* proved to be structurally unisexual at the individual level in the analyzed populations due to the primary sexual dimorphism identified. This characteristic has been previously observed and described in other studies—e.g., [42,43]. Moreover, we identified the presence of vestigial reproductive organs of the opposite sex in both female (staminodes) and male (pistillodes) flowers. This had also been described in the species [38,42,43], as well as in several species of the genus *Diospyros* [44,45,46] and of the Ebenaceae family [7,36]. This evidence confirms that *D. sericea* is structurally dioecious. However, we also identified variations, such as fruit development in what should be structurally male individuals. These individuals are predominantly male with occasional hermaphroditic flowers.

In terms of secondary dimorphisms, the species has a wide variation in phenology between its areas of occurrence. In Brazil, flowers and fruits were recorded throughout the year, varying according to the region of occurrence [42]. Flowering synchronicity between the sexes is essential for the reproductive success of dioecious species [47]. However, male and female plant strategies may differ from one another to favor the pollen flow [10,15,48]. For example, we observed that, in *D. sericea*, female inflorescences are reduced to a single flower which probably also allows a better resource supply. Another common characteristic is that the flowering period of female individuals tends to be shorter, allowing greater resource allocation and energy provision for fruit and seed development [15,48,49].

The flowering of male individuals tends to happen earlier [50,51,52], over a longer period, and with a higher flower production [53,54]. Flowering intensity among the male flowers was nearly twice that observed among the female flowers, which could be explained by the higher number of flowers per axil found in male individuals. This trait, frequent among other dioecious species [47], has been previously identified in *D. sericea*—e.g., [37,55]—as well as the whole family Ebenaceae [36]. 

The flowering phenophases occur in *D. sericea* throughout the whole year, which possibly induces constant pollinator visits [56,57]. Since the majority of sexually dimorphic dioecious plants are pollinated by animals [15], their reproductive success relies on these pollinating agents [15,58]. Thus, as well as depending on a certain degree of flowering synchronicity between the sexes, dioecious species (including *D. sericea*) also depend on the phenology of floral visitors. This relationship is particularly advantageous if the flowering peaks coincide with periods of higher pollinator abundance [15]. Asynchronous flowering peaks between the sexes, which we observed in *D. sericea*, may favor outcrossing for reducing competition for flower visitors [57,59]. Altogether, these data suggest that the reproductive success of this dioecious species lies beyond its own conditions and phenological traits, depending directly on the pollinator’s presence.

In terms of floral morphology, male flowers tend to be smaller than female ones as a common pattern in dioecious species, which also occurs in the family Ebenaceae and the genus *Diospyros* [36,38]. However, this characteristic is not uniform, as we can see in *D. sericea*. We observed morphological similarities in size and general physical aspects between pistillate and staminate flowers. Both female and male flowers were inconspicuous and barely specialized, which are frequent traits among dioecious species [15,30,32,60]. These morphological similarities, along with differences in resource availability offered by male and female flowers (e.g., pollen), may enable pollination by deceit [61]. When male and female flowers are similar and a given resource is available in only one of the sexes, pollinators may visit both staminate and pistillate flowers [61,62,63,64]. Thus, considering the morphological similarities between its unisexual flowers and the differences in resource supply between the sexes, pollination by deceit may be common in *D. sericea* flowers.

In species whose female flowers have an anticipated flowering peak among the male flowers, this works as a strategy for pollinator deceit. Pollinators visit the staminate flowers in search for pollen and eventually visit pistillate flowers, where they fail to find this resource [61]. This strategy seems successful in *D. sericea*, where female flowers do not produce pollen but are visited for deceit probably due to the morphological similarity between female and male flowers. 

The presence of scent in flowers of both sexes is also important for the reproduction of dioecious species, given that olfaction is one of the most used senses by insects [65,66]. In this case, scent plays the role of a distance attraction resource [67,68,69], a mechanism that also contributes to automimicry and which has been reported in other dioecious species [15,70,71]. The typical scent of *D. sericea*, although also present in female flowers, is stronger in male flowers, as also observed in other dioecious species—e.g., [72]. This may promote greater efficiency in pollen transport due to higher attractiveness to pollinators. 

Other than the pollinator attraction strategies, *D. sericea* also seems to have adapted to the environmental conditions towards maximizing the success of its reproductive cycle until its final stage, which is seed dispersal. Female individuals have the highest flower production at the end of the dry season, as also described in *D. lasiocalyx* [73]. This phenological behavior does not seem ideal for these individuals, given that female reproduction is costly, and water is limited during this period. However, according to [74], plants can overcome limitations in water availability through mechanisms that avoid the effects of drought on reproduction. One of these mechanisms was observed in *D. sericea*, whereby defoliation in female individuals only increased significantly between the dry and the rainy seasons, which coincided with the flowering peak and the most intense fruiting phase. This trait suggests nutrient relocation from senescing leaves to fruit formation [75]. Another favorable phenological adaptation is fruit development during more humid months, a common feature among animal-dispersed species [76] (birds and primates consume fruits and disperse seeds of *D. sericea* [42]). An advantage associated with this is that water availability favors the production of fleshy fruits, which become more conspicuous to seed dispersers [77], increasing the likelihood of reproductive success.

The onset of fruiting in female flowers before anthesis completion may indicate high efficiency in male reproductive strategies for providing large amounts of pollen, coupled with pollinator efficiency. However, in this case, apomixis also cannot be ignored. Considered a type of asexual reproduction, apomixis is the formation of seeds without fertilization, which can result in embryos developed from the ovule tissues [78,79]. The facultative apomictic species reproduce sexually but may produce seeds without fertilization as an alternative path to reproduction [78]. *D. sericea* fruiting traits and dependency on pollinators, coupled with the common observation of apomixis in its genus [80,81], could suggest the possibility of the species showing facultative apomixis, however we have not found evidence for apomixis in the studied species. 

The analysis of *D. sericea* floral biology calls the evolutionary process of its reproductive system into consideration. A possible evolutionary pathway to dioecy involves gynodioecy, reviewed by Ashman [82]. Since a gynodioecious population is composed of female plants and monoecious ones [12] or plants with female and hermaphroditic flowers [20,83], autogamy (i.e., self-pollination) could be possible, not depending directly on pollinating agents. Despite fertilization taking place before anthesis completion in *D. sericea*, no pollen was found in female flowers’ staminodes; thus, autogamy is unlikely unless the female flowers showed occasional well-developed staminodes with viable pollen, which we did not find. 

Fruit development in predominantly male individuals with occasional hermaphroditic flowers of *D. sericea* suggests a direct link with the presence of vestigial female reproductive organs in its flowers. According to Wallnöfer [36], structurally male flowers in the Ebenaceae family bear pistillodes that are seldom absent and sporadically well-developed. Considering this trait, an evolutionary pathway that passes through androdioecy is also possible. Androdioecy is considered an intermediate sexual type in the evolutionary process towards dioecy, defined by the occurrence of populations formed by monoecious individuals [12] or by individuals with perfect flowers (i.e., hermaphroditic) and others with sterile female sexual functions [20,83]. Thus, the fact that its reproductive organs are highly viable from the beginning of floral development may allow self-fertilization in male flowers bearing pistillodes that, for a random evolutionary transition cause, are occasionally functional, leading to fruit formation in male individuals.

Another evolutionary pathway would be the inexpression of the female sex and/or intensification of the male functions in hermaphroditic ancestors, allowing resource relocation from female to male functions to evolve and resulting in subdioecious populations [14]. These are normally considered dioecious, with occasional occurrence of monoecious individuals [14,21]. In subdioecious populations, male individuals are considered “inconstant” due to their ability of occasional seed production. Thus, with occasional manifestations of monoecy in *D. sericea* populations, some individuals that appear predominantly male would be capable of producing fruits, even if at a low frequency. This could be the most probable evolutionary pathway for dioecy in *D. sericea*.

## 4. Materials and Methods

### 4.1. Study Site and Model Plant Species

The study was performed in the Serra Nova district, Rio Pardo de Minas municipality, Minas Gerais state, Brazil. The data were collected in the surroundings of the Parque Estadual de Serra Nova e Talhado (PESNT) (coordinates 42°57′30″ W and 16°1′30″ S), which has a total area of 49,890 hectares.

The climate in the region falls in the category BSh, according to the Köppen climate classification (hot, semi-arid climate), with average precipitation between 380 and 760 mm and an average annual temperature above 18 °C. According to the climate data collected in the municipality of Rio Pardo de Minas between 1983 and 2005 [84], the average temperature in the region is 22.5 °C, oscillating between 19.3 and 24 °C. The rainy season occurs between November and March, representing 85.4% of total annual precipitation, and peaks in December (200 mm). The dry season occurs between May and September, representing 3.84% of total annual precipitation, and peaks in August (2 mm).

The PESNT is located in a transitional region between the Cerrado and Caatinga phytogeographic domains in a landscape intersected by rocky outcrops from the northern Espinhaço mountain range. Grasslands, rocky grasslands (Campos Rupestres), cerrado savannas, cerrado grasslands, semideciduous seasonal forests (gallery forests), and deciduous seasonal forests (dry forests) compose the PESNT vegetation [84]. 

The selected plant species was *Diospyros sericea*, a widely distributed species in Brazil, occurring in the Amazon, Caatinga, Cerrado, Atlantic Forest, and Pantanal phytogeographic domains [85]. It has a tree or shrub habit, and its main human use is for building houses [43,86]. Although edible, its fruits are seldom consumed by humans [87] but are highly appreciated by birds and primates. The latter, particularly, play an essential role in *D. sericea* seed dispersal. *D. sericea* is well distributed in the surroundings of the PESNT, especially in abandoned pastures and monocultures under natural regeneration. Wood extraction is frequent and indiscriminate on the site.

### 4.2. Phenology

#### 4.2.1. Data Collection

We selected 17 adult male and 13 adult female individuals (at reproductive age) of *D. sericea* with completely visible canopies to monitor phenology. When possible, we ensured a distance of 5 m between individuals aiming to capture greater genetic variability and lower relatedness among them [88]. 

We recorded phenological data every month for one year between September 2015 and August 2016. To identify the vegetative phenophases (leafing and defoliation), we adapted the methodology proposed by [89]. Leafing was identified as the period between initial bud development and the formation of young leaves. Defoliation was identified as a change in leaf color and subsequent fall. The reproductive phenological events that we observed were flowering and fruiting. We subdivided the flowering phase into initial flowering (flower buds) and established flowering (flowers in anthesis). We subdivided the fruiting phase into initial fruiting (immature fruits) and established fruiting (mature fruits). 

#### 4.2.2. Data Analysis

We observed the crown of each individual to record the phenological observations, estimating the total manifestation of each phenological event. We followed Fournier [90] to analyze phenological data. We also estimated phenological synchronicity in the population, following Morellato et al. [91]. 

We used our observations on the development and persistence of crown leaves to classify the species into 4 phenological groups (according to [92]): (1) evergreen with continuous growth (ECG), without evident deciduousness and leaf production during long periods; (2) evergreen with seasonal growth (ESG), without total deciduousness but with leaf replacement in the transition between the dry and rainy periods; (3) brevi-deciduous (BDC), with total deciduousness in the dry season for a period shorter than two weeks; and (4) deciduous (DEC), with total deciduousness in the dry season for a period longer than two weeks.

### 4.3. Floral Biology

For the floral biology analyses, we collected one flower from 10 male and 10 female individuals. These analyses were conducted in the Laboratory of Plant Ecology and Systematics at the Federal Institute of Northern Minas Gerais—Salinas campus.

We assessed floral development in situ by marking and observing one pre-anthetic flower of ten different individuals (n = 10) during the morning, afternoon, and night for seven days (totaling 54 h). Pre-anthesis was considered as the period when flowers were fully formed but with a closed corolla.

#### 4.3.1. Morphometric Analyses

We measured the floral features of randomly selected male (n = 26) and female (n = 26) flowers. Two or three flowers were collected from ten different plants of each sex. We used a digital caliper with a precision of 0.1 mm to measure the studied traits. The floral features analyzed were the length and diameter of the calyx, corolla, and pistil, and the number and length of stamens. We also characterized the distribution and number of buds per axil in male and female individuals.

#### 4.3.2. Pollen Integrity and Stigma Receptivity

Pollen release was assessed in situ from flowers (n = 10) in pre-anthesis, open flowers (n = 10), and senescing flowers (n = 10). The pollen viability test was done in pre-anthetic flowers (n = 10) and open flowers (n = 10) belonging to ten different structurally male individuals. For this, the anthers were macerated and stained with acetic carmine (1.2%) in a semipermanent slide [93]. The slides were analyzed under a light microscope (Nova Optical Systems, 180i) with an objective lens of 10× magnification, and the first 200 pollen grains observed were manually counted. We considered viable the pollen grains that changed color to reddish pink and inviable the pollen grains that did not change color at all. Pollen quantity was expressed in percentage terms.

Stigma receptivity was tested in situ in flower buds (n = 10), pre-anthesis flowers (n = 10), and open flowers (n = 10). We applied hydrogen peroxide at 3% (H_2_O_2_) to the stigmas [94] and, with a magnifying glass (10×), interpreted the formation of bubbles as a sign of respiratory activity and therefore stigma receptivity.

#### 4.3.3. Scent

We assessed the presence and type of floral scent by placing recently opened flowers inside glass jars that were later sealed for 20 min [94]. We used separate containers for male and female flowers. Then, we analyzed the odor intensity by the human sense of smell from three different individuals. To detect floral scent glands (osmophores), we submerged the flowers for five minutes in neutral red solution (1:1000) and washed them in running water [70].

### 4.4. Statistical Analyses

All data were analyzed in BioEstat 5.0. We compared the morphometric measurements between male and female flowers (floral features and number of flower buds per leaf axil) with a Student’s *t*-test (*p* ≤ 0.05). In a few cases, when the requirements for the *t*-test were not met, the Mann–Whitney U test was used (*p* ≤ 0.05). The results of the pollen integrity test at different floral developmental stages were analyzed with a one-way ANOVA.

We calculated Spearman correlations (𝑟𝑠; 5% of significance) between the phenological phases of leafing, defoliation, initial flowering (flower buds), established flowering (flowers in anthesis), initial fruiting (immature fruits), and established fruiting (mature fruits), and the climatic variables during the sampling months (average temperature, relative humidity, and accumulated precipitation). The climate data (Figure 1 and Figure 2) were drawn from the website of Brazil’s National Institute of Meteorology—INMET [95], specifically from the automatic weather station from Rio Pardo de Minas, Minas Gerais state

## 5. Conclusions

*D. sericea* exhibits the common features of a dioecious species in all aspects analyzed. Primary sexual dimorphism is evident, whereas secondary sexual dimorphism mainly appears in phenological traits and floral attractants and resources. However, female and male flowers are morphologically similar.

Importantly, vestigial reproductive organs were generally found in flowers of both sexes, which is common among *Diospyros* species. This trait may be associated with occasional fruit development in predominantly male individuals with occasional hermaphroditic flowers, characterizing them as inconstant males

The strong morphological similarity between male and female flowers and the existence of staminodes/pistillodes together with the occasional fruiting of male flowers suggests that dioecy in this species is of recent origin. Although our data do not explain the causes of fruit formation in predominantly male individuals with occasional hermaphroditic flowers, our descriptions may be a preliminary step for future analyses on the evolution of dioecy in *D. sericea*. The production of seeds exclusively by females was already pointed out as a handicap of dioecy [96]. Due to the general absence of fruit production in males, female individuals should produce twice as much seeds [20], a problem that may be attenuated in *D. sericea* due to fruit production in males. In this case, seed production is adding to other traits associated with the success of dioecious species such as precocious reproduction and animal-dispersed fleshy fruits, among others [97]. The promising information presented in this study calls attention to the need for a phylogenetic-based comparative approach to compare species in the *Diospyros* genus. Thus, perhaps it will be possible to unveil the enigma of the origin and establishment of dioecy in *D. sericea* and in the genus *Diospyros*. 

The comprehension of dioecious species’ sexual system, such as we found in *D. sericea*, is essential to its conservation and the entire ecosystem in which it interacts with pollinating and seed-dispersing animals. In this particular case, the variations in the expression of the sexual system requires that, for conserving a dioecious species, beyond focusing on male and female individuals, it would also be essential to identify matrices that represent the sexual transition, such as inconstant males. Thus, in a broader perspective, these studies may contribute to the conservation of this species and dioecious species in general. By taking into account the morphological variation, the conservation will be preserving not only a species but its ecological and evolutionary history.

## Figures and Tables

**Table 1:** Spearman correlation between the climatic variables (average temperature (°C), relative air humidity (% UR), and precipitation (mm); recorded between September 2015 and August 2016) and the intensity of vegetative (leafing and defoliation) and reproductive (buds, flowers, immature and mature fruits) phenophases of individuals of *D. sericea*.

Phenology	Individuals	Phenophases	Average Temperature	Relative Humidity	Precipitation
**Vegetative**	Male	Leafing	-	-	−0.16
		Defoliation	0.75 *	−0.81 *	0.21
	Female	Leafing	-	-	-
		Defoliation	0.56 *	−0.75 *	0.16
**Reproductive**	Male	Initial flowering	−0.27	-	0.002
		Established flowering	−0.08	−0.02	0.18
		Initial fruiting	0.31	−0.15	0.50
	Female	Initial flowering	−0.40	0.04	−0.02
		Established flowering	−0.05	0.03	0.27
		Initial fruiting	0.52	−0.43	0.16
		Established fruiting	0.91 *	−0.60 *	0.47

Initial flowering (flower bud); established flowering (flowers in anthesis); initial fruiting (immature fruit); established fruiting (mature fruit). * Indicates significant differences (*p* ≤ 0.05) according to the Spearman correlation analysis (rs).

**Table 2:** Mean, standard deviation and variation in the morphometric measurements of floral whorls (centimeters), the number of stamens/staminodes, and the number of buds per axil (units) of individuals of *D. sericea* PESNT. $\bar{x}$ = mean; SD = standard deviation.

Structures Measured	Male		Female		*t-Test or U Test*	*p*
	$\bar{x}\;$± SD	Min-Max Range	$\bar{x}\;$± SD	Min-Max Range		
**Calyx**						
Length	0.77 ± 0.077	0.59–0.94	0.80 ± 0.053	0.71–0.90	*t* = −1.96	0.0549
Diameter	0.45 ± 0.046	0.32–0.53	0.53 ± 0.038	0.44–0.59	*t* = −6.42	<0.0001 *
**Corolla**						
Length	1.13 ± 0.107	0.98–1.35	1.09 ± 0.075	0.95–1.21	*t* = 1.88	0.0655
Diameter	0.43 ± 0.054	0.30–0.55	0.48 ± 0.050	0.34–0.59	*t* = −3.30	0.0018 *
**Pistil/pistillodes**						
Length	0.12 ± 0.087	0.06–0.52	0.68 ± 0.083	0.39–0.79	*t* = −23.62	<0.0001 *
Diameter	0.13 ± 0.043	0.08–0.23	0.33 ± 0.022	0.30–0.41	*U* = 0.00	<0.0001 *
**Stamens/staminodes**						
Length	0.62 ± 0.056	0.49–0.72	0.43 ± 0.042	0.35–0.50	*t* = 13.47	<0.0001 *
Number	43.07 ± 4.811	34–54	9.35 ± 3.908	4–15	*U* = 0.00	<0.0001 *
**Number of buds *per* axil**	2.18 ± 0.566	1–7	1	1	-	-

* Indicates significant differences (*p* ≤ 0.05) according to a *t*-test or a Mann–Whitney test.

**Table 3:** Pollen integrity (%) tested in flower buds, flowers in pre-anthesis, and open flowers of *D. sericea* from individuals located in the surroundings of PESNT.

Floral Development Stage	Min–Max Range	$\bar{x}\;$± SD	Pollen Integrity (%)
Flower buds	163–197	182.4 ± 12.72	91.2%
Flowers in pre-anthesis	167–197	186.0 ± 9.043	93%
Open flowers	177–197	187.7 ± 6.667	94%
